# *In vitro* Biocompatibility of New Silver(I) Coordination Compound Coated-Surfaces for Dental Implant Applications

**DOI:** 10.3390/ma4020355

**Published:** 2011-01-28

**Authors:** Priscilla S. Brunetto, Tünde Vig Slenters, Katharina M. Fromm

**Affiliations:** University of Fribourg, Department of Chemistry, Chemin du Musée 9, CH-1700 Fribourg, Switzerland

**Keywords:** silver, cytotoxicity, biocompatibility

## Abstract

Biofilm formation on implant materials causes a common problem: resistance to aggressive pharmacological agents as well as host defenses. Therefore, to reduce bacterial adhesion to implant surfaces we propose to use silver(I) coordination networks as it is known that silver is the most powerful antimicrobial inorganic agent. As a model surface, self-assembled monolayers (SAMs) on gold Au(111) was used to permit permanent attachment of our silver(I) coordination networks. The surface coatings showed typical nano-structured surfaces with a good biocompatibility for soft-tissue integration with fibroblast cells.

## 1. Introduction 

Foreign-body materials are used more and more frequently in our current life: joint implants (hips, knees, fingers, *etc.*), catheters, pacemakers, dental and aesthetic implants, *etc.* Biofilm formation, a well-developed bacterial survival strategy against pharmacological agents as well as host defenses, causes serious implant-related infections [1,2,3,4]. Adhesion of bacteria to dental implant components and restorative materials is a prerequisite for the formation of a biofilm that may lead to the development of dental diseases like dental caries, periodontal diseases and peri-implantitis [5,6]. 

One way to prevent, or at least diminish the bacterial adhesion to implants is to render their surface bactericidal. The current revival of silver chemistry in this context initiated us to use this metal ion for coating purposes. Indeed, silver is used in a wide range of applications for antimicrobial surfaces in the field of medical surgery [7,8,9,10,11]. To prevent infections associated with indwelling and percutaneous medical devices, manufacturers are increasingly looking to silver as the solution. Thus, the objective is to modify the surfaces of devices in such a way that they become a safe, biocompatible, non-toxic defense against bacteria and fungi (including antibiotic resistant bacteria). Experiments with classical silver salts have lead to unsatisfactory properties. Coordination polymers containing silver cations are promising candidates for surface modification as they exhibit i) high structural stability, ii) little decomposition upon light exposure, and iii) low solubility in aqueous media [12,13,14,15,16,17]. 

Biocompatibility and biosafety are considered to imply that the clinical application of a biomaterial should neither cause any adverse reaction nor endanger the life of the patient. The attachment of anchorage cells is the first step in the process of cell-surface interactions which can affect cellular and tissue responses. Cell-surface interactions remain complex and poorly understood, largely because of the vast diversity of processes and parameters which control the adhesion. Both the microtopography and the surface chemistry of the substrate are known to exert an influence on cell-surface interactions. Surface chemistry is known to influence cellular adhesion. 

In this work, we have studied the *in vitro* biocompatibility of silver-coated surfaces by analyzing the influence of silver-treatments of gold implant surfaces on fibroblast growth. Cytotoxicity and cell physiological aspects were evaluated and compared with the results obtained using the uncoated gold surfaces normally used in dental practice.

## 2. Results and Discussion

### 2.1. Coating and Surface Characterization

**Figure 1:**
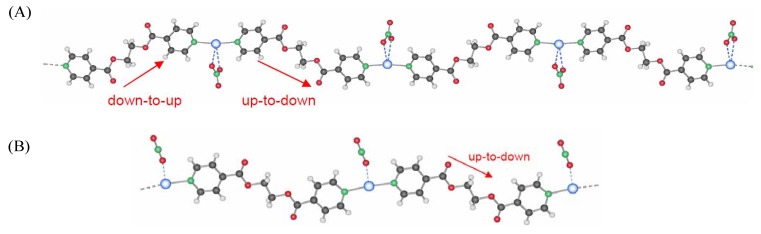
Structure of [Ag(L)NO_3_]_n_, (**A**) compound **1**, (**B**) compound **2** [12,13,14,15,16,17,18]; Color code: Ag: blue; O: red; N: green; C: grey; H: white.

As shown earlier [18], we are able to control the synthesis of different silver coordination polymer compounds based on L = ethanediyl bis(isoniconate), such as the two polymorphs of [Ag(L)NO_3_]_n_, **1** and **2** (Figure 1). These compounds exhibit a high structural stability but silver(I) compound **2** showed the best light stability without degradation upon light exposure and a low solubility in aqueous media. 

As a model substrate for the deposition of silver(I) compound **2**, an oriented gold monolayer Au(111) on glass, was used. The specimens were first cleaned and pre-treated with a disulfide derivative of our ligand and then immersed into the mother liquor (EtOH-THF) of 2 mM solution of silver(I) compound **2**. To characterize the coating obtained, powder X-ray, topography and wettability measurements were used as it is known that surface characterization is essential for correlating any surface modification with changes in biological performance. Indeed, surface properties can have an enormous effect on the success or failure of a biomaterial device.

#### 2.1.1. Powder X-ray diffraction

**Figure 2:**
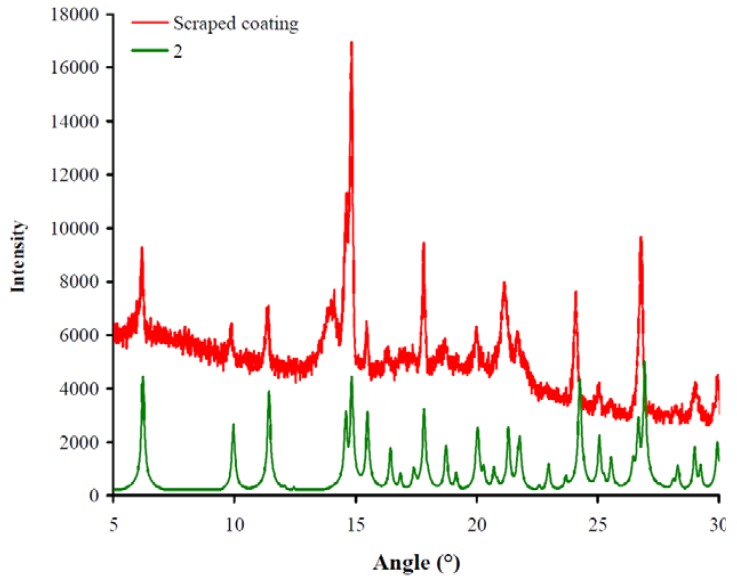
X-ray powder diffraction spectra of silver(I) compound onto the modified gold surface. The scraped coating (in red) was compared with the silver(I) compound **2** from the literature (in green).

Powder X-ray diffraction is the most reliable method to identify silver(I) compounds coated on gold Au(111) surfaces. An X-ray powder diffraction of the compounds scraped off from the modified gold Au(111) surfaces (Figure 2, shown in red) was measured and compared to the simulated spectra from the single crystal data of silver(I) compounds **1** and **2**. Figure 2 shows together a spectrum of the silver(I) compound **2** (in green) and the scraped coating (in red). 

#### 2.1.2. Topography 

**Figure 3:**
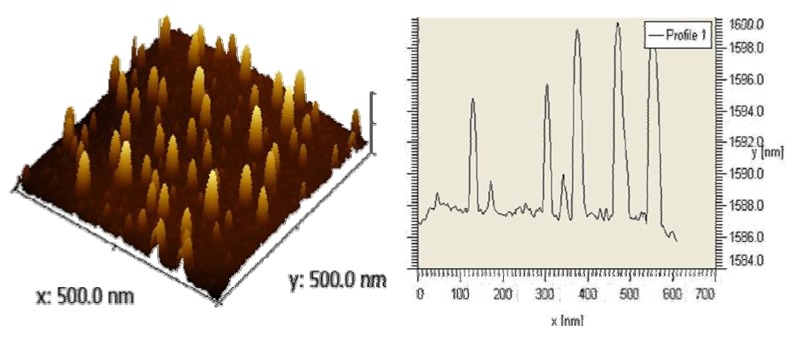
Atomic Force Microscopy (AFM) picture and profile of the deposit of compound **2** on Au(111) [21].

**Figure 4:**
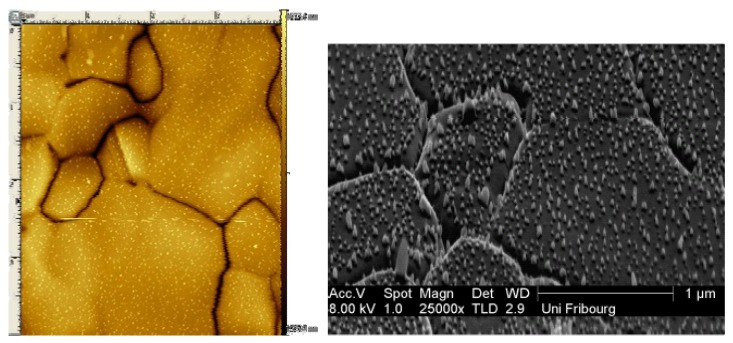
AFM (left) and scanning electron microscopy (SEM; right) pictures of the deposit of compound **2** on Au(111) [21].

The success of using microscopic methods to characterize biomaterial surfaces is well established [19,20]. Atomic Force Microscopy (AFM) investigations of gold Au(111) coated with silver(I) compound revealed a peak-like structure on the surface, a pattern that corresponds to Ostwald ripening motif with a main interpeak distance of 20-30 nm (Figure 3 left). From the mean peak height of 11-14 nm, the average polymer chain length supposed to be standing upright on the surface indicates octameric chains of [Ag_8_L_7_], while the width correspond to 200-400 chains linked together within one peak for silver(I) compound **2**. This was confirmed by scanning electron microscopy measurements (Figure 4). Short treating times favor formation of a robust nano-structure, while longer treating times result in a denser and thicker coverage of the surface with formation of brittle aggregates. It is therefore recommended to have a good nano-structured coating rather than large crystals on the surface. The concentration dependency is evident, as at low concentrations, the time to reach a regular nano-structured coating takes longer than at higher concentrations [21,22].

#### 2.1.3. Surface wettability

For our purposes, one of the critical factors influencing adhesion of cells onto a substratum is the wettability of the biomimetic materials. The wettability (generally referred to as hydrophobicity/hydrophilicity) can be estimated by determining the contact angle of liquid droplets. A drop of fluid is placed on the biomaterial surface of interest at an equilibrium position and the contact angle (θ) is determined from the tangent associated with the drop/surface. Typically, in the biomaterials literature, contact angles are measured in air, utilizing a goniometer, using the sessile drop method with water as the test fluid. Contact angle analysis of control and modified materials can quickly provide valuable information about the relative hydrophilicity/hydrophobicity of the surfaces. By definition, a surface can be considered hydrophilic when the angle θ < 90°, whereas for a hydrophobic surface angle θ > 90°.

To determine if our newly silver coated surfaces were hydrophobic or hydrophilic surfaces, we measured the contact angle for water sessile drops on gold Au(111) surfaces. Uncoated gold (that serves as a control) and surfaces coated with silver(I) compound were analyzed. Mean contact angles for silver(I) compound coatings was 66.1° when tested with distilled water. A mean contact angle of 65° was observed for the gold uncoated surface. 

Wettability depends on the roughness of the surface [23] and contributes to the cellular response [24]. Moreover, wettability seems to play a dominant role especially in the initial period of cell-material interaction [25,26,27]. In the literature, hydrophobic non-wettable surfaces are undesirable due to adherence of bacteria and adsorption of protein. Generally, hydrophobic surfaces are considered to be more protein-adsorbent than hydrophilic surfaces because of the strong hydrophobic interactions occurring at these surfaces [28,29,30], in direct contrast to the repulsive solvation forces arising from strongly bound water at the hydrophilic surface [31]. Protein adsorption on the implant surfaces initiates the biofouling response leading to the failure of implanted medical devices within one month of implantation. Therefore, wettable surfaces are required for many biomaterials. Indeed, as shown in [32,33,34,35], cells have been observed to adhere, spread and grow more on moderately wettable surfaces with water contact angles of 50-70°. 

In our case, all coated-surfaces were hydrophilic. These coatings showed promising effects in antimicrobial studies [36] and can be suitable surfaces for biocompatibility, as discussed later.

### 2.2. Antimicrobial Properties

Our new surfaces modified by silver(I) compound **2** were tested for their bactericidal activity as it is known that silver compounds possess antimicrobial properties. The antimicrobial activity was first assessed by micro- and nano-calorimetry, showing that bacterial growth is strongly delayed in the presence of our silver compounds [36]. The *in vitro* Live/Dead Assay investigations in a flow chamber, imitating the oral environment [21], showed that bacteria (*S. epidermidis)* are killed upon contact with our substrate. The antimicrobial activity was also investigated by the well known Kirby-Bauer test. Both tested Gram-positive strains, *S. aureus* and *S. epidermidis*, mainly responsible for the implant related infection, and Gram-negative bacteria *E. coli* and *P. aeruginosa* were susceptible against our silver coated surfaces [36]. The inhibition zones reached up to 1.5 cm in diameter, which is a signature of a very efficient antimicrobial agent. We assumed that the biofilm is rather not protective against silver. Furthermore, we demonstrated that the compound has a curative effect upon *S. epidermidis* infection in a murine implant infection model [36].

### 2.3. Cell Growth and Viability Evaluation

Fibroblasts are strongly involved in the wound healing process at the implant’s soft tissue interface. Therefore, it is crucial that these cells arrive prior to the bacteria at the implant surface where they can cover the surface and make it less vulnerable to bacterial colonization.

**Figure 5:**
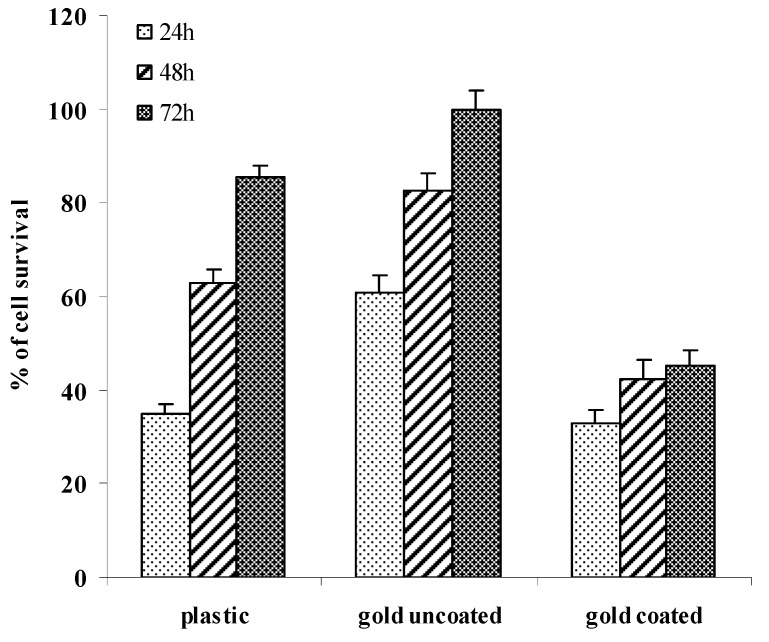
Effect of compound **2** on oriented gold surfaces. Cell viability was assessed at 24, 48, and 72 h of culture.

Mouse fibroblast cells were used as a cell model to investigate the effects of surface and material variations on soft tissue response. Our silver(I) compound **2** coated on gold surfaces showed a good antimicrobial activity, and to address the biocompatibility an MTT assay was performed for the cell proliferation and by implication cell cytoxicity. MTT results, assayed after 24, 48 and 72 hours of culture, are shown in Figure 5. As a control, cells were grown on plastic and gold Au(111) uncoated surfaces. Each assay was carried out in triplicate for statistical evaluation. 

Cells grew well on uncoated gold oriented Au(111) surface coated with silver(I) compound **2** as compared to plastic surfaces after three days of culture (Figure 5). Gold surfaces coated with a thin layer of our silver(I) compound **2** showed a lower cell response with up to 45% cell survival. Fibroblast cells still slowly increased after three days of culture (Figure 5). Moreover, *in vivo* investigations in a murine implant infection model showed that our silver-coated surfaces allowed a slow and controlled silver release, which limited leukocyte cytotoxicity and caused minimal host cell damage [36]. Our new silver-coated surfaces were biocompatible and were promising for further *in vitro* experiments. 

### 2.4. Morphological Examination

**Figure 6:**
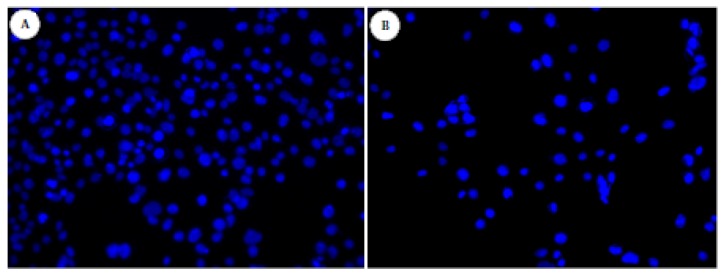
Effect of silver(I) compound coated-surfaces on fibroblast cells. DAPI staining was assessed at day 3. Cells were fixed onto surfaces, permeabilized, stained with DAPI and examined using a fluorescent microscope. (**A**): gold Au(111) uncoated; (**B**) gold Au(111) coated with silver(I) compound **2**.

**Figure 7:**
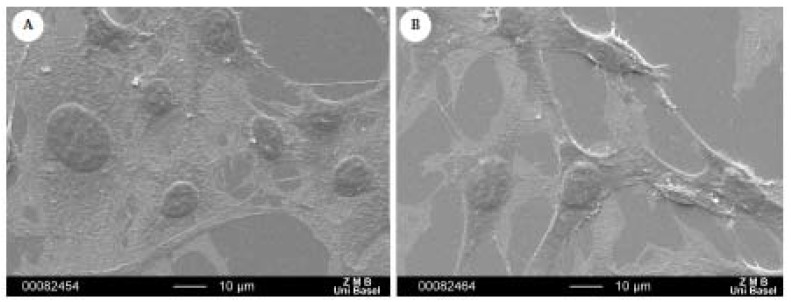
Effect of silver(I) compound coated-surfaces on fibroblast cells. Cell proliferation was assessed at day 3. Cells were fixed onto surfaces, dehydrated, thinly sputted with gold and examined using a Scanning Electron Microscope. (**A**): gold Au(111) uncoated; (**B**) gold Au(111) coated with compound **2**.

When cultured on surfaces, the total area that the fibroblasts spread on their surface correlates with the amount of cell adhesion on that surface, and promotes cell proliferation and colonization [37,38] Hence, cell spreading and adhesion give an indication of the cytocompatibility of different experimental substrates *in vitro*. Fibroblast morphology on the oriented gold surfaces was evaluated *in vitro.* The cytotoxicity assays gave us an idea of the biocompatibility of our new silver(I)-coated surfaces. The same gold Au(111) surfaces coated with silver(I) compound **2** was tested for cell morphology using DAPI staining and SEM techniques (Figure 6 and Figure 7, respectively). When DAPI binds to DNA, its fluorescence is strongly enhanced, which has been interpreted as a highly energetic and intercalative type of interaction. Because of this property, DAPI is a useful tool in various cytochemical investigations for observing if there are adherent cells or not.

Fibroblast cells were observed on non-treated gold Au(111) surfaces, that served as control (Figure 6, left). Among the modified gold surfaces, cells could be observed on gold surfaces treated with compound **2** (Figure 6, right).

The results obtained with DAPI staining were confirmed by scanning electron microscopy (Figure 7). Fibroblasts were observed on the treated gold surfaces with silver(I) compound **2**. Fibroblasts were generally well spread on both coated surfaces. These results confirmed what we previously observed with the cytotoxcity test. 

## 3. Experimental Section 

### 3.1. Surfaces

The gold surface has a size of about 11 × 11 mm. The gold monolayer on glass was flame annealed to obtained the Au(111) terraces. The flame annealing was made with a Bunsen burner. As the flame annealing removes all impurities, there is no need for further cleaning. We used a disulfide derivative of our ligand to graft our silver(I) polymer and our antibiotic derivative. Since it is know that the S–S bond of the disulfide is broken when a bond forms between the S atoms and the Au, the SAM was formed by placing the gold surface into a solution of ethanol and dichloromethane (1:1) that contained 5 mM disulfide for 7 days. The SAM modified surfaces were thoroughly rinsed with ethanol upon removal from the disulfide deposition solutions and then placed onto a mixture of our targets. After removal from the disulfide solution and the wash step, the SAM modified surfaces were placed into a mixture solution containing our ligand L1 and silver nitrate at 2 mM concentration dissolved in THF/EtOH (1/1) each. After 3 hours incubation time, our test specimens were rinsed three times with pure ethanol and dried over P_2_O_5_ until their use.

### 3.2. Characterization of Surface Topography

#### 3.2.1. X-Ray Powder Diffraction (XRPD)

Powder diffraction for all samples was measured on a *STOE STADI P automated diffractometer*, with CuK source, graphite monochromator, by using quartz sample holders; for the pure reagent, 10-25 mg of substance was used. The program PowderCell 2.2 was used for calculation of X-ray powder patterns on the basis of single crystal structure determinations. Experimental and calculated patterns were then compared.

#### 3.2.2. Microscopy techniques

Scanning Electron Microcopy (SEM) images were measured in two different modes namely: contrast or back-scattering modus depending on the sample. Some EDS measurement were done using the same device. For Atomic Force Microscopy (AFM), all experiments were performed on a dry sample at room temperature using a semi-contact (or Tapping mode) AFM.

#### 3.2.3. Wettability

To characterize surface wettability, contact angle analysis was performed on all surface modifications. The sessile drop method was used for contact angle measurement with a commercial contact angle meter. For every surface modification, three measurements (each with at least five drops of distilled water) were made at room temperature to provide adequate replications for statistical analysis.

### 3.3. Fibroblast Cell Culture

3T3 fibroblast murine cell lines (ATCC) were used as a cell model to investigate the effects of surface and material variations on soft tissue response. The fibroblast cultures were maintained in DMEM supplemented with 10% fetal bovine serum, 2% of L-Glutamate and 0.5% penicillin/streptomycin at 37 °C in humidified air and 5% CO_2_. Cultures were subdivided by trypsination using Trypsin-EDTA solution. The modified gold surfaces were placed in standard 24-well tissue culture plates (Nunc), and cells were then seeded. As a control substrate for cell attachment and growth, fibroblasts were plated directly onto tissue culture polystyrene plastic. The culture medium was changed every 3 days and cells were incubated for 3 days at 37 °C in humidified air and 5% CO_2_.

### 3.4. MTT Assay of Fibroblast Cells

A quantitative colorimetric MTT test was performed after 1, 2 and 3 days of culture to characterize cellular metabolism (vitality) and, by implication, proliferation. The test specimens were pre-incubated in DMEM/10% fetal bovine serum for 20 hours at 37 °C, then washed with PBS and placed in new 24-wells plates. Cells were seeded at the right density onto the tests specimens. At day 1, 2 and 3, the tests specimens were placed in new wells and 150 μL of fresh medium was added. 50 μL of MTT solution [5 mg/mL 3-(4,5-dimethylthiazol-2-yl)-2,5-diphenyl-tetrazolium bromide in PBS] was added to each well, and the cells were incubated at 37 °C for 4 h. The reaction was stopped at 4 °C for hours. The medium was then removed and 500 μL of dimethylsulfoxide was added to each well, followed by 30 min incubation at room temperature on a shaker. One hundred and fifty microliters of each solution was transferred in a 96-well plate. The optical density (OD) was measured at 540 nm with an ELISA Reader. The mean absorbance values were corrected for a blank (medium only) and results were reported as optical density.

### 3.5. Cell Spreading and Morphology

#### 3.5.1. Fluorescence microscopy of fibroblast cells (DAPI staining)

The test specimens were pre-incubated in DMEM/10% fetal bovine serum for 20 hours at 37 °C, then washed with PBS and placed in new 24-well plates. Cells were seeding at the right density onto the tests specimens. At day 3, the tests specimens were placed in new wells. Fibroblasts were rinsed with PBS 0.1 M at pH 7.0 to remove the non-adherent cells and then fixed with 2% formaldehyde solution (in PBS) for 10 min. The specimens were washed with PBST three times for 10 min at room temperature and DAPI dye diluted in PBST (1:1000) was added for 20 min staining. After a final rinse with PBST, cells were examined and photographed using a fluorescence microscope. Fibroblasts on gold and tissue culture polystyrene plastic served as controls.

#### 3.5.2. Scanning electron microscopy (SEM) 

The test specimens were pre-incubated in DMEM/10% fetal bovine serum for 20 hours at 37 °C, then washed with PBS and placed in new 24-well plates. Cells were seeded at the right density onto the tests specimens. At day 3, fibroblasts were rinsed with phosphate-buffered saline (PBS) to remove the non-adherent cells and then fixed with 2% formaldehyde solution for 10 min. After a final rinse with PBS and water, cells were dehydrated through a graded series of ethanol from 30%, 50%, 70%, 90% and 100%. After air drying, surfaces were thinly sputter coated with platinum. Fibroblasts on gold and tissue culture polystyrene plastic served as controls.

## 4. Conclusions

We have presented here a silver(I) compound coated on a metallic gold surface showing a robust nano-structured coating, which it is therefore recommended for the biocompatibility. These silver-coated surfaces were hydrophilic with a good antimicrobial effect on bacterial biofilm formation *in vitro* as well as *in vivo*. The materials were tested for their biocompatibility with fibroblast cells and turned out to be the ideal candidates for such antimicrobial coatings. Indeed, we could show via SEM, MTT and DAPI staining tests that fibroblasts are i) alive on our substrates and ii) proliferate as well. Our silver(I) compound coated surface is a promising candidate for further analysis *in vitro* for hard-tissue integration experiments.
